# *In vivo* and *in vitro* Pathogenesis and Virulence Factors of *Candida albicans* Strains Isolated from Cutaneous Candidiasis

**DOI:** 10.29252/ibj.24.5.319

**Published:** 2020-02-19

**Authors:** Golnar Sadeghi, Seyed Fazllolah Mousavi, Mina Ebrahimi-Rad, Esmat Mirabzadeh-Ardekani, Ali Eslamifar, Masoomeh Shams-Ghahfarokhi, Zahra Jahanshiri, Mehdi Razzaghi-Abyaneh

**Affiliations:** 1 Department of Medical Mycology, Pasteur Institute of Iran, Tehran 1316943551, Iran;; 2 Department of Microbiology, Pasteur Institute of Iran, Tehran 1316943551, Iran;; 3 Department of Biochemistry, Pasteur Institute of Iran, Tehran 1316943551, Iran;; 4 Department of Medical Biotechnology, Pasteur Institute of Iran, Tehran 13164, Iran;; 5 Department of Clinical Research, Pasteur Institute of Iran, Tehran 13164, Iran;; 6 Department of Medical Mycology, Faculty of Medical Sciences, Tarbiat Modares University, Tehran 14115-331, Iran

**Keywords:** Candida albicans, Experimental candidiasis, in vivo pathogenicity, Virulence factors

## Abstract

**Background:**

The *Candida albicans *is one of the most important global opportunistic pathogens, and the incidence of candidiasis has increased over the past few decades. Despite the established role of skin in defense against fungal invasion, little has been documented about the pathogenesis of *Candida* species when changing from normal flora to pathogens of vaginal and gastrointestinal epithelia. This study was carried out to determine the *in vivo* and *in vitro* pathogenesis of clinical *C. albicans* strains isolated from skin lesions.

**Methods:**

In this study, association of *in vivo* and *in vitro* pathogenesis of *C. albicans* isolates with different evolutionary origins was investigated. Oral and systemic experimental candidiasis was established in BALB/C mice. The expression levels of *SAP1-3* genes, morphological transformation, and biofilm-forming ability of *C. albicans* were evaluated.

**Results:**

All the strains showed *in vitro* and *in vivo* pathogenicity by various extents. The *SAP1*, *SAP2, *and *SAP3* genes were expressed in 50%, 100%, and 75% of the strains, respectively. The biofilm formation ability was negative in 12% of the strains, while it was considerable in 38% of the strains. Fifty percent of the strains had no phospholipase activity, and no one demonstrated high level of this pathogenesis factor. Relatively all the strains had very low potency to form pseudohyphae.

**Conclusion:**

Our findings demonstrated that *Candida albicans* strains isolated from cutaneous candidiasis were able to cause oral and systemic infections in mice, so they could be considered as the potential agents of life-threatening nosocomial candidiasis in susceptible populations.

## INTRODUCTION

The polymorphic fungus *Candida albicans* belongs to the normal flora of human mucosa and in most individuals is considered as a non-pathogenic commensal. Under certain conditions, *C. albicans* can, however, cause infection that ranges from superficial, such as cutaneous and oral, to life-threatening systemic candidiasis. The worldwide incidence of candidiasis has increased over the past few decades, which may be attributable to the increased numbers of immunocompromised patients^[^^1 ▶^^,^^2 ▶^^]^. Infection by *Candida* yeasts has been shown to be endogenous in origin. However, nosocomial infections are generally transmitted by the health care staff or relatives’ hands, from patient to patient, or even by medical devices^[^^3 ▶^^]^. Despite the established role of skin in defense against fungal invasion, little has been documented about the pathogenesis of *Candida* species when invading vaginal and gastrointestinal surfaces. *C. albicans* is the major causative agent of superficial *Candida*-related infections and causes skin thickening, crusting, inflammation, and erythema^[^^4 ▶^^-^^6 ▶^^]^. Although laboratory experiments provide reliable data on the virulence and pathogenesis of *Candida*-related infections, the data from host-fungal pathogen interaction will be quit important to clarify how the fungus affects the human tissue and invade the skin of various body parts^[^^7 ▶^^,^^8 ▶^^]^. 

 Amongst the rodents, mice are the most widely used animal models for investigating clinical *Candida* isolates from oral mucosa or systemic candidiasis. Such types of candidiasis have been induced experimentally in outbred and inbred mice strains. The most commonly used inbred mice strains are BALB/C^[^^9 ▶^^,^^10 ▶^^]^. It assumes that the ability of *C. albicans* to be a pathogen from commensal results from producing various virulence factors. In this relation, the morphological transition between yeast and pseudohyphae (known as dimorphism), production of secretory enzymes i.e. Saps, and phospholipase and formation of biofilm are significant virulence factors contributed to *C. albicans* pathogenesis^[^^11 ▶^^,^^12 ▶^^]^. 

 The present study was designed to evaluate the ability of molecular-typed *C. albicans* strains^[^^13 ▶^^] ^isolated from skin lesions to induce systemic and oral candidiasis in murine models. In our study, the presumptive relation between *in vivo* and *in vitro* pathogenesis and differences in molecular types and evolutionary origin of *C. albicans* isolates were considered.

## MATERIALS AND METHODS

**Study population and sample collection**

Eight clinical *C. albicans* isolates, as the cause of cutaneous candidiasis, typed by MLST were studied. A portion of the specimens was inoculated on SDA plates (Merck, Germany) supplemented with chloramphenicol (0.05 gL ^-1^) and incubated at 37 °C for 24 h, to isolate the *Candida* species. All the isolates were identified by a combination of morphological and biochemical analysis including germ tube formation, chlamydospore formation, carbohydrate assimilation (using the commercial system ID32 C,biomérieux Marcy l’Etoile, France), and CHROMagar *Candida* screening (CHROMagar *Candida*, Paris, France). Species identification was confirmed by internal transcribed spacer sequencing. 

**Animal model**

Isogenic female BALB/C mice (n = 120, 6-8 weeks old) were obtained from Pasteur Institute of Iran (Tehran, Iran). The mice were kept in plastic cages, allowed free access to water and maintained on a 12:12 h light/dark cycle. The temperature and humidity were controlled at 23 ± 1 °C and 55 ± 10%, respectively. A number of 56 mice were divided into eight groups of seven mice with different *in vitro* pathogenesis for each of the oral and systemic candidiasis. In each experiment, four mice were considered as the negative controls. 

**Experimental systemic candidiasis**

The mice were challenged by the intravenous injection of an inoculum of 2 × 10^7^ CFU in 100 µl of a yeast suspension from cultures grown on SDA via a 25-gauge syringe at 37 °C for 48 h. The mice were observed for seven days, and the mortality was noted. For CFU determination in organ, the mice were sacrificed by cervical dislocation after seven days. Kidneys were aseptically harvested as most of the *Candida* cells in systemic candidiasis murine models are found in the kidneys^[^^14 ▶^^]^. Each was divided into equal portions for fungal burden determination and histopathological evaluation. For fungal burden determination, the kidneys were weighed and then homogenized in 1 ml of PBS, and homogenates were serially diluted before plating on SDA. The plates were incubated at 37 °C for 24 h, and the fungal burden was expressed as the ratio of log_10_ of CFU to the organ weight^[^^15 ▶^^]^. 

**Experimental oral candidiasis**

The murine model of oral candidiasis was set up according to a formerly described method with some modifications^[15,16]^. Establishment of infection at mucosal sites generally requires treatment with immunosuppressive agents prior to infection. One day before infection, the mice were immunosuppressed by subcutaneous injection of prednisolone (100 mg/kg body weight), and the antibiotic tetracycline hydrochloride (0.83 mg/ml) was dispensed in the drinking water. Before infection, the mice were anesthetized with 100 µl of ketamine (100 mg/kg of mouse)/xylazine (10 mg/kg of mouse) administered intraperitoneally via intramuscular injection on each femur. The oral cavities of the anesthetized mice were swabbed with a sterilized cotton swab that had been dipped in a cell suspension of each strain of *C. albicans* (2.0 × 10^7^ CFUs/ml from cultures grown on SDA at 37 °C for 48 h). The mice were sacrificed 72 hours after infection. The tongues were divided into equal portions for fungal burden determination and histopathological evaluation. For fungal burden determination, the tongue was weighed and then homogenized in 1 ml of PBS, and homogenates were serially diluted before plating on SDA. The plates were incubated at 37 °C for 24 hours, and fungal burden was expressed as the ratio of log_10_ of CFU to the organ weight^[^^16 ▶^^]^. 

**Histopathological study**

For histopathological analysis, kidneys and tongues were fixed in 4% paraformaldehyde, fixed with paraffin wax and stained with periodic acid-Schiff. 

**RNA isolation**

Total RNA from *C. albicans* grown in culture was prepared using the RNeasy RNA isolation system, the Molecular Biology kit (Bio Basic Inc, Canada) according to the manufacturer’s instructions and stored at -70 °C until use. RNA concentration was measured by the absorbance at 260 nm. 

**Synthesis of cDNA**

To synthesize the cDNA, PrimeScript First strand cDNA Synthesis Kit (Takara Bio Inc, Japan) was used according to the instructions recommended by the manufacturer. 

**Real-time PCR**

Real-time PCR was used to quantify tity r the virulence genes (*SAP*1-3) mRNA levels in our isolates, with *ACT1* used as a reference housekeeping gene. The PCR primers for amplification of *SAP *and *ACT1* were previously described by Monroy-Pérez *et al.*^[^^17 ▶^^] ^(Table 1). A final volume of 25 µl, including 12.5 µl of SYBER Green Master Mix, 1 µl of each forward and reverse primer, 2 µl of cDNA (20 ng), and 8.5 µl of RNase-free water, was used for each reaction. The amplification conditions were performed with an initial denaturation of 95 °C for 10 minutes, followed by 35 cycles of 20 s at 95 °C, 40 s at annealing temperature of 58 °C, and 30 s at 72 °C. The gene expression was evaluated by the ΔC_T_ method using the control gene (*ACT1*) to normalize the data. Each reaction was performed in triplicate, and the mean values of relative expression were analyzed for each gene^[18]^. The *C. albicans* ATCC10231 strain was used as a positive control. The strains from our collection, each carrying a different deletion of *SAP* genes, were used as the negative controls^[^^10 ▶^^]^. 

**Virulence factors**

The *in* *vitro* biofilm formation of *Candida* isolates was determined as described by Zago *et al.*^[^^19 ▶^^]^. The metabolic activity of the biofilm was measured by crystal violet (1% v/v) assay after 48 hours of incubation. Based on the average OD observed, the isolates were classiﬁed into three groups showing: (i) low (OD less than 0.12), (ii) medium (OD of 0.12–0.20), and (iii) high (OD higher than 0.20) bioﬁlm-forming. Phospholipase activity was evaluated by the methods mentioned by Tsang *et al.*^[^^20 ▶^^]^ and Galan-Ladero *et al.*^[^^21 ▶^^]^. Suspension of 10^8^ cell/ml was dropped onto each test medium. After 48 hours of incubation at 37 °C, the diameters of the colonies (a) and the diameters of the precipitation zone around the colonies (b) were measured. The production of the enzyme was designated as Pz = a/b. The following ranges of activity according to Pz index were established: high, Pz = 0.40; medium, Pz = 0.41–0.60; low, Pz = 0.61–0.80; very low, Pz = 0.81–0.99; none, Pz = 1. The Pz index is a reproducible semi-quantitative technique used widely. Pseudohyphae formation was deﬁned as a cell bearing a rounded outgrowth with a length greater than or equal to the diameter of the parent cell, with a constriction at the base. After two hours of cell growth in a liquid medium containing an equal volume of RPMI 1640 (Sigma, USA) and fetal bovine serum (GIBCO, USA), the percentage of cells in pseudohyphae form against blastopores was determined by microscopy, counted as described by Negri *et al*^[^^22 ▶^^]^.

**Table 1:** Primers used in real-time PCR assay

**Gene**	**Primers**	**Sequences (5'-3')**	**Size of amplified product (bp)**
*SAP1*	*SAP*1F	TCAATCAATTTACTCTTCCATTTCTAACA	161
	*SAP*1R	CCAGTAGCATTAACAGGAGTTTTAATGACA	
*SAP*2	*SAP*2F	AACAACAACCCACTAGACATCACC	178
	*SAP*2R	TGACCATTAGTAACTGGGAATGCTTTAGGA	
*SAP*3	*SAP*3F	CCTTCTCTAAAATTATGGATTGGAAC	231
	*SAP*3R	TTGATTTCACCTTGGGGACCAGTAACATTT	
*ACT*1	*ACT*1F	GACCGAAGCTCCAATGAATC	181
	*ACT*1R	AATTGGGACAACGTGGGTAA	

**Statistical analysis**

Results were analyzed using the Graph Pad Prism 6. One-way ANOVA and Unpaired *t*-test were used for the statistical analysis. *p* values <0.05 were considered statistically significant.

**Ethical statement**

The above-mentioned sampling/treatment protocols were approved by the Institutional Ethics Committee of Pasteur Institute of Iran (ethical code: IR.PII.REC. 1397.021).

## RESULTS

**Experimental systemic and oral candidiasis**

**Fig. 1:**
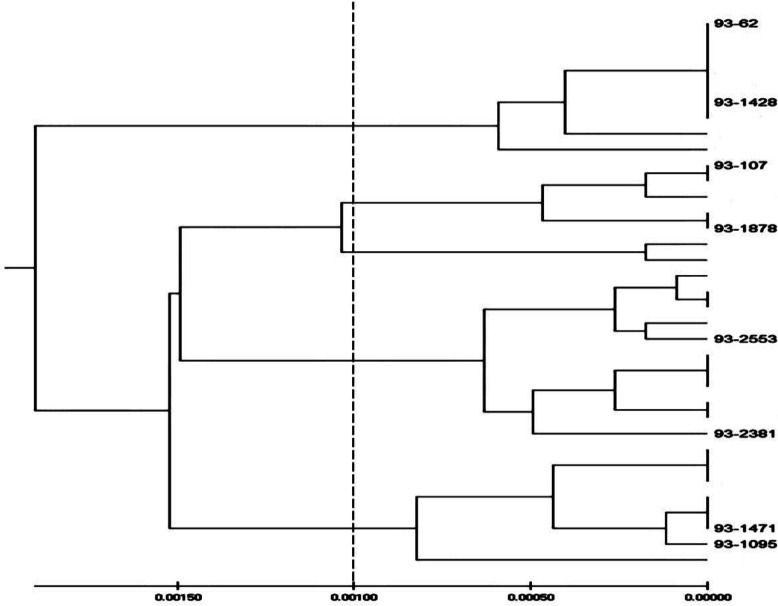
The dendrogram constructed from UPGMA analysis based on concatenated MLST sequence of the seven loci of eight  *C. albicans* strains

The present study was designed to evaluate the virulence activity of the *C. albicans* strains isolated from skin lesions to induce systemic and oral candidiasis in murine models. Eight *C. albicans* isolates were selected according to the highest and the lowest *in vitro* virulence activity in four clades derived from typing by MLST method^[^^13 ▶^^] ^(Fig. 1). Each of two isolates of separate minor clades showed different *in vitro* and *in vivo* pathogenicity, but there was no evident association between the strains in variant major clades and all studied forms of pathogenesis. 

***SAP*** **expression**

**Fig. 2:**
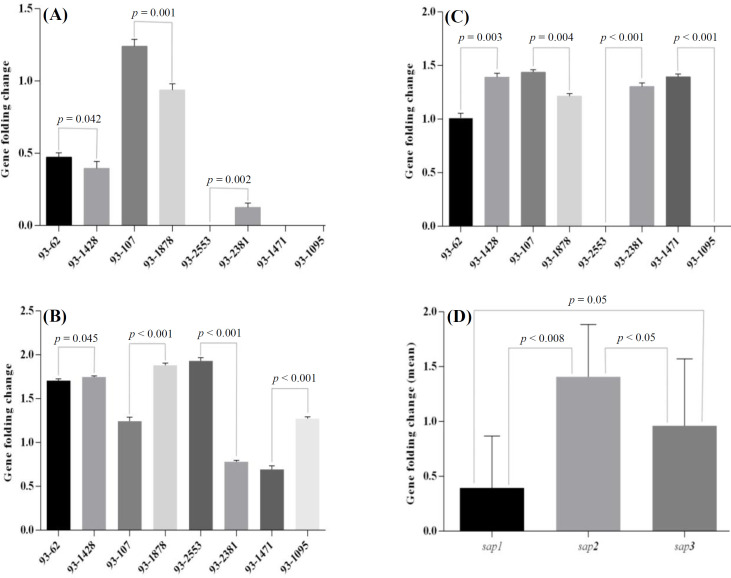
Comparison of *SAP1-3* expression according to the folding change (2^-ΔΔCT^) in eight *C. albicans* isolates. *SAP1* (A), *SAP2 *(B), *SAP3* (C), and the mean values of *SAP1-3* expression (D).

The primers used in real-time PCR assay are shown in Table 1. Each of the different *SAP* primer sets were specified for each *SAP* gene, excluding the possibility that any one *SAP* primer set could cause a false-positive result. We examined the expression of genes encoding Sap1-3 in eight *C. albicans* isolates. Each of the *SAP1*, *SAP2*, and *SAP3* genes was expressed in 50%, 100%, and 75% of the strains, separately. Three patterns in the genotypes of *SAP1-3* were identified. Four strains expressed all the studied *SAP* genes, two isolates *SAP2* and *3* and two isolates only one. The folding changes (2^-ΔΔCT^) of the *SAP1*-*3* genes in all the isolates from different minor clades with their mean values are illustrated in Figure 2. 

**Virulence factors**

Table 2 displays the *in vitro* virulence ability such as *SAP* expression, biofilm formation, phospholipase production, and pseudohyphae creation. The biofilm formation rates in 12% of the isolates were negative and in 38% high. Also, 50% of the strains had no phospholipase activity, and no one demonstrated the high level of this pathogen factor. Relatively, all the strains had low capability to form pseudohyphae. 

**Histology in relation to virulence**

**Fig. 3:**
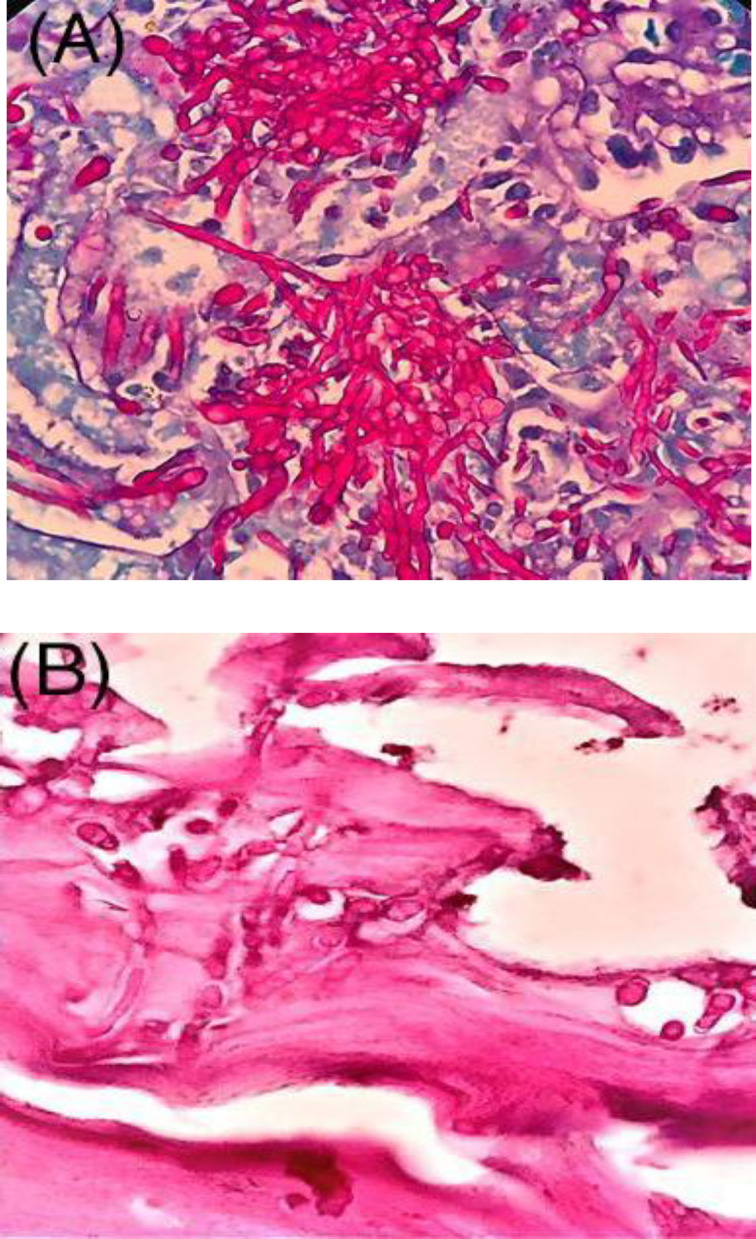
Microscopic observation of typical lesions on the kidneys and tongues of mice with systemic and oral candidiasis on days 7 and 3 after the infection, respectively. Periodic acid-Schiff-stained sections show the extensive colonization by numerous *Candida *hyphae in the kidney (A) and on the epithelium of the dorsal surface of the tongue (B). (×1000 magnification)

Weights of mice and organs, mortality, and *in vivo* pathogenicity in animals challenged with eight *C. albicans* isolates to form oral and systemic infections demonstrated in Table 3 were different. All of these strains isolated from patients suffering from cutaneous candidiasis with different pathogenic potential were able to cause systemic and oral candidiasis in mice. Histopathological analysis of kidneys and tongues, collected from BALB/c mice in which systemic and oral candidiasis were induced, showed different incidence of established infection and inflammation by *C. albicans *fitting to their *in vivo* pathogenesis*,* with abundant yeasts and the formation of pseudohyphae/ hyphae (Fig. 3). 

## Discussion

Candida infections are one of the most significant nosocomial infections that have proliferated by 3.5 to 14 folds over the past decades. Although the sources of infection are human normal flora, hospital environments have an undisputable role ^[^^23 ▶^^]^. During both superficial and systemic infection, C. albicans strains depend on a complex of virulence factors. It has been shown that the injury of host epithelial cells induced by filamentous forms (hyphae and pseudohyphae) of C. albicans is the main factor facilitates the growth and invasion of the fungus ^[^^24 ▶^^]^.

Our results showed that in contrast to morphological transition, enzymatic activities were not involved in fungal pathogenesis. These findings are in accordance with the results of a previous study implying that a high percentage of phospholipase production (53.8- 74%) by C. albicans isolates ^[^^25 ▶^^]^. In another study, authors showed that Candida isolates from candidemia has considerable phospholipase activity contributed to their virulence. Despite the known role of filamentous forms of C. albicans in fungal pathogenesis, yeast cells of the fungus were abundantly reported from infected tissues ^[^^26 ▶^^]^. It has been demonstrated that biofilm- forming ability was more frequent among bloodstream isolates than that of the isolates from other clinical sources ^[^^27 ▶^^]^. Other virulence factors of C. albicans in oral candidiasis are closely related to molecular factors such as Saps, in particular Sap1-3, whereas in denture stomatitis, biofilm formation is the most important factor ^[^^28 ▶^^]^. To date, different roles are attributed to Saps (level of activity and number of SAP genes) of C. albicans in relation to pathogenesis, which mainly facilitate the fungal invasion and affect host immune responses ^[^^29 ▶^^]^. 

**Table 2:** *In vitro* pathogenicity in eight different *C. albicans* isolates

**No.**		***SAP *** **expression**				**Biofilm** ^a^	**Phospholipase** **(P**_z_ **)** ^b^	**Pseudohyphae** **(%)** ^c^
		***SAP1***	***SAP2***	***SAP3***				
93-62		+	+	+		+2	0.42	8
93-1428		+	+	+		-	1.00	5
93-107		-	+	+		+2	1.00	5
93-1878		+	+	+		+3	0.84	29
93-2553		-	+	-		+1	1.00	4
93-2381		-	+	+		+3	0.66	17
93-1471		-	+	+		+2	1.00	6
93-1095		-	+	-		+3	0.70	11

(-) negative; ^a^1+, 2+, and 3+: %Tbloc (Measure of light blocked when passing through the wells) 5-20, 20-35, and 35-50, respectively. ^b^P_z _= the diameters of the colonies (A) and the precipitation zone (B) around the colonies (medium: Pz = 0.41–0.60, low: Pz = 0.61–0.80, very low: Pz = 0.81–0.99, and none: Pz = 1). ^c^The percentage of cells in pseudohyphae form. Seven mice were challenged for each of the isolates.

Our findings showed that each of the *SAP1*, *SAP2*, and *SAP3* genes was expressed in 50%, 100%, and 75% of the strains, separately. *SAP2 *and* 3* expressions in our study were similar to a previous report for *C. albicans* strains with oral and vaginal source by Dabiri *et al.*^[^^18 ▶^^] ^in Iran, whereas the result for *SAP1 *activity was different. According to a previous report by Lima* et al.*^[^^30 ▶^^]^, the *SAP1* gene had more expression in the process of vaginal infection in comparison to the oral infection. More investigation suggested that *SAP1-3 *appears to be essential for mucosal and *SAP4-6* for systemic infections^[^^31 ▶^^]^. Our results demonstrated that the *SAP2 *gene can be considered as an effective pathogenic factor for tissue destruction in oral candidiasis. As indicated in another investigation, *SAP2* gene may be a potential target for prevention or treatment of candidiasis^[^^32 ▶^^]^.

**Table 3:** Weights of mice and organs, mortality, and *in vivo* pathogenicity in animals challenged with eight *C. albicans* isolates to form oral and systemic infections

**No.**	**Mouse weight change (g)** **mean (range)**		**Organ weight (g)** **mean (range)**		**Mortality ** **(day)**		***In vivo*** ** pathogenicity (Log CFU/g tissue) (median) range**	
	**Systemic**	**Oral**	**Systemic**	**Oral**	**Systemic**	**Oral**	**Systemic**	**Oral**
93-62	-3.9 ((-5.2)-(-2.4))	- 4.0 ((-5.3)-(-2.6))	0.21 (0.14- 0.30)	0.07 (0.04- 0.09)	2/7 (5)	0/7(0)	(4.43) 3.69- 4.73	(4.41) 2.95- 5.26
								
93-1428	-1.5 ((-2.5)-(-0.7))	-4.2 ((-5.5)-(-2.8))	0.20 (0.12- 0.28)	0.07 (0.05- 0.10)	0/7 (0)	1/7(2)	(1.75) 1.03- 2.78	(4.73) 4.59- 4.82
								
93-107	-1.0 ((-2.3)-(-0.2))	-3.8 ((-5.6)-(-0.5))	0.18 (0.14- 0.20)	0.07 (0.04- 0.09)	1/7 (7)	0/7(0)	(2.67) 1.46- 4.33	(4.22) 3.87- 4.55
								
93-1878	-5.1 ((-7.2)-(-2.6))	-4.2 ((-5.2)-(-3.1))	0.20 (0.16- 0.24)	0.07 (0.05- 0.10)	0/7 (0)	0/7(0)	(3.37) 2.39- 4.35	(4.72) 4.30- 5.27
								
93-2553	-3.9 ((-6.0)-(-0.3))	-4.5 ((-5.5)-(-3.5))	0.19 (0.18- 0.22)	0.07 (0.05- 0.09)	1/7 (5)	0/7(0)	(3.89) 3.23- 4.44	(5.11) 4.13- 5.68
								
93-2381	-2.5 ((-4.3)-(-0.7))	-4.1 ((-5.2)-(-2.8))	0.19 (0.14- 0.22)	0.08 (0.03- 0.11)	0/7 (0)	1/7(2)	(3.68) 1.93- 4.97	(4.81) 4.38- 5.19
								
93-1471	-5.2 ((-8.7)-(-3.3))	-4.0 ((-5.3)-(-2.9)	0.20 (0.14- 0..21)	0.09 (0.07- 0.11)	0/7 (0)	0/7(0)	(3.59) 2.75- 4.79	(4.64) 4.32- 5.29
								
93-1095	-4.4 ((-4.8)-(-3.5))	-4.5 ((-5.3)-(-2.8))	0.19 (0.17- 0.21)	0.09 (0.07- 0.10)	1/7 (5)	0/7(0)	(3.75) 2.48- 5.28	(5.19) 4.98- 5.54

We have experimented oral and systemic models of candidiasis using *C. albicans* strains isolated from skin lesions and selected four clades derived from typing by MLST method^[^^13 ▶^^]^. The murine model that is considered as the “gold standard” in the study of oral and systemic *Candida* infections can be used to prove genetic determinants of *Candida* virulence ability^[^^32 ▶^^]^. Although the source of the fungal isolates may be a neglected confounding factor in virulence studies in animal models, in our study, all the isolated strains from patients with skin lesions were able to cause systemic and oral candidiasis in mice. Each of two isolates of separate minor clades demonstrated various degree of comparable pathogenicity, but there was no evident association between strains in variant major clades and all studied forms of pathogenesis. For instance, strains of 93-62 and 93-1428 in a minor clade clearly vary in a number of properties not exactly related to the particular gene sequences that are used to express their clade assignation. A similar report to our study was presented by MacCallum *et al.*^[^^33 ▶^^]^. It has been shown that phenotypic amendment in *C. albicans* changes gradually over the years as a result of small alterations in the sequences of some genes^[^^34 ▶^^]^. In agreement with Tavanti *et al.*’s^[^^35 ▶^^]^ report, the utility of MLST for determining clade assignments of clinical isolates will form a basis for the rational selection of a wider diversity of strains for future researches into *C. albicans* virulence. It is obvious that either fungal characteristics or host reactions determine the outcome of fungal infection and development of clinical manifestation. Although the importance of hypha production and phospholipase production in *Candida* pathogenicity is undisputed, the yeast can also disseminate and multiply in internal organs. Besides, our results, in a few isolates, indicated that other factors may account for the observed variation in the development of infection in animals (Table 2). Presumably, the ability to cause diseases in the mammalian host is the product of a combination of these phenotypes and others. This observation was similar to a previous report by Asmundsdóttir *et al.*^[^^36 ▶^^]^. Ideally, for meaningful comparison, many representative isolates should be used. 

Taken together, our findings further indicate that virulence factors of *C. albicans* are important determinants of the outcome of *Candida*-related invasion in relation to phenotypic and genotypic characteristics. Inclusive comparative studies from different geographical areas of the world are highly recommended in order to make a global profile of virulence in *C. albicans*.
